# Design of an Electromagnetic Micro Mirror Driving System for LiDAR

**DOI:** 10.3390/s24123969

**Published:** 2024-06-19

**Authors:** Jie Chen, Haiqiang Zhang, Zhongjin Zhang, Wenjie Yan

**Affiliations:** 1 Key Laboratory of MEMS of Ministry of Education, Southeast University, Si-Pai-Lou 2, Nanjing 210096, China; 220221739@seu.edu.cn (H.Z.); 220211696@seu.edu.cn (W.Y.); 2 Sgmicro, Building C, No. 2167-2 Qunli Second Avenue, Daoli District, Harbin 150000, China; 15946430515@163.com

**Keywords:** electromagnetic micro mirror, PLL, driving system

## Abstract

Electromagnetic micro mirrors are in great demand for light detection and ranging (LiDAR) applications due to their light weight and low power consumption. The driven frequency of electromagnetic micro mirrors is very important to their performance and consumption. An electromagnetic micro mirror system is proposed in this paper. The model of the system was composed of a micro mirror, an integrated piezoresistive (PR) sensor, and a driving circuit was developed. The twisting angle of the mirror edge was monitored by an integrated PR sensor, which provides frequency feedback signals, and the PR sensor has good sensitivity and linearity in testing, with a maximum of 24.45 mV/deg. Stable sinusoidal voltage excitation and frequency tracking was realized via a phase-locked loop (PLL) in the driving circuit, with a frequency error within 10 Hz. Compared with other high-cost solutions using PLL circuits, it has greater advantages in power consumption, cost, and occupied area. The mechanical and piezoresistive properties of micro mirrors were performed in ANSYS 19.2 software. The behavior-level models of devices, circuits, and systems were validated by MATLAB R2023a Simulink, which contributes to the research on the large-angle deflection and low-power-consumption drive of the electromagnetic micro mirror. The maximum optical scan angle reached 37.6° at 4 kHz in the behavior-level model of the micro mirror.

## 1. Introduction

As a light detection system, light detection and ranging (LiDAR) can achieve high-resolution, high-precision ranging, speed measurement, and precise object sensing functions. LiDAR has the advantages of long detection distance, good coherence, and high spatiotemporal resolution, which makes it widely used in fields such as robotics, autonomous driving, security, and environmental monitoring [1]. The micro mirror, as a micro-opto-electromechanical system (MOEMS) device, has been widely used in many applications, such as medical, automotive, consumer, and military electronics [2,3,4], etc. Meanwhile, there is a growing demand for small LiDAR with broad research prospects [5]. A vast quantity of micro mirrors using electrothermal [6], electrostatic [7], piezoelectric [8], and electromagnetic [9] actuation have been developed in the past. Electromagnetic micro mirrors have received wide attention because of their small size, large deflection angle, low driving voltage, and low power consumption [10,11,12]. Wang [13] designed a new type of LiDAR, with a MEMS reflector aperture of 1.2 mm × 1.4 mm, capable of scanning a field of view of 9° × 8°. Ye [14] proposed an electromagnetic micro scanning mirror based on a titanium alloy with a large aperture of 12 mm and a fast-scanning frequency of 1.24 kHz. When the driving current is 250 mApp, the optical scanning angle can reach 26° at the resonant frequency.

The electromagnetic resonance micro mirror is playing a growing role in the research on LiDAR, while the influence of air and structural damping causes significant energy dissipation. So, the quandaries of the micro mirrors lie in the domains of power consumption and angular deflection. Circuit or algorithm modulation can be used to achieve open-loop driving of micro mirrors. Primary excitation of micro mirrors can be achieved through fixed-frequency signal excitation [15,16,17]. The scanning range and vibration continuity are limited by the open-loop driving due to the damping of the system.

Automatic gain circuit (AGC) and phase-locked loop (PLL) are two common types of circuits for MEMS resonators to achieve closed-loop driving [18]. Liao [19] proposed a closed-loop adaptive control scheme for the precise positioning and trajectory tracking of electrostatically driven torsion mirrors. Compared with open-loop control and the PID controller, this scheme has better performance in step response and trajectory tracking and can compensate for parameter changes of the micro mirror online. In the experiment, ±1.3° scanning was achieved at 23.44 kHz. Brunner [20] proposed a novel digital PLL with a position-sensitive detector (PSD), a charge-coupled device (CCD), and a continuous laser source implemented in FPGA. The circuit was operated at a 100 MHz internal clock to realize 100 Hz and 28.76° scanning with a setting time of approximately 88.9 ms. The proposed sensing method carries the potential for high-precision and high-speed scanning control of MOEMS mirrors oscillating with several thousand hertz. An online Hammerstein-model-based predictive optimization control (POC) was designed by Cao [21] using online estimated parameters and model residuals to achieve high-precision angle positioning in a noisy environment, and its stability and effectiveness were verified. To improve the angular positioning accuracy, a piecewise PID control based on the filters’ design for this electromagnetic micro mirror was proposed by Sun [22]. The design of the FPGA and PSD controllers improved the micro mirrors’ stability and scanning performance. However, the system consisting of the crystal oscillator, PSD, and ADC circuits increases chip area and system complexity [23]. All these efforts expressed the necessity of a low-consumption and compact micro mirror system to optimize the LiDAR system.

**Figure 1:**
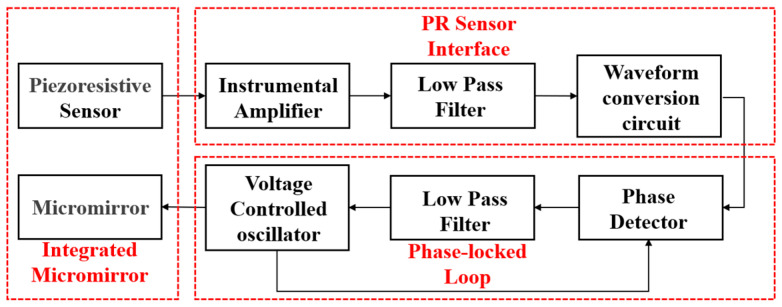
The block of the electromagnetic micro mirror with an integrated PR sensor.

An electromagnetic micro mirror system was proposed to improve the scanning performance. The system block diagram proposed is shown in Figure 1. A micro mirror device with an integrated PR sensor was designed. The PR sensor, highly doped, was located at the edge of the supporting beam to realize the torsion angle detection of the micro mirror. The output of PR sensor was followed by an instrumental amplifier, the low pass filter, and waveform conversion circuits. The PR interface circuit adjusted the waveform and amplitude of the PR sensor output signal. The circuit composed of a PLL circuit and waveform conversion circuits was used to drive the electromagnetic micro mirror. The basic PLL consists of a phase detector (PD), a low-pass filter (LPF), and a voltage-controlled oscillator (VCO). The system can also reduce the complexity and chip area occupied, increasing the possibility of low-power and lightweight design. It achieves high-speed and high-precision scanning, and, meanwhile, it saves resources. Simulation of the driving system, which is highly non-linear, was achieved using MATLAB Simulink and was applied to the electromagnetic micro mirror. This paper is focused on the modeling of the micro mirror driving system. The finite element model of the micro mirror with an integrated PR sensor was developed in ANSYS. Meanwhile, the model of the driving circuit and micro mirror at the behavioral level was developed in MATLAB Simulink.

## 2. Model and Theory

In the micro mirror system, a sinusoidal voltage was generated by a VCO with an initial frequency of 3500 Hz to drive the micro mirror, and the detection of the torsion angle was achieved by the PR sensor during the vibration. When there was a deviation between the excitation signal frequency and the natural frequency of the system, the feedback signal of the micro mirror will shift towards the natural frequency point. The feedback signal generated by the PR sensor was compared with the VCO output signal through the PD. The phase difference was amplified, and a DC control voltage was generated via the LPF. The micro mirror system has feedback between the micro mirror and the driving circuit, so the resonant drive of the micro mirror due to self-oscillation was realized.

**Figure 2:**
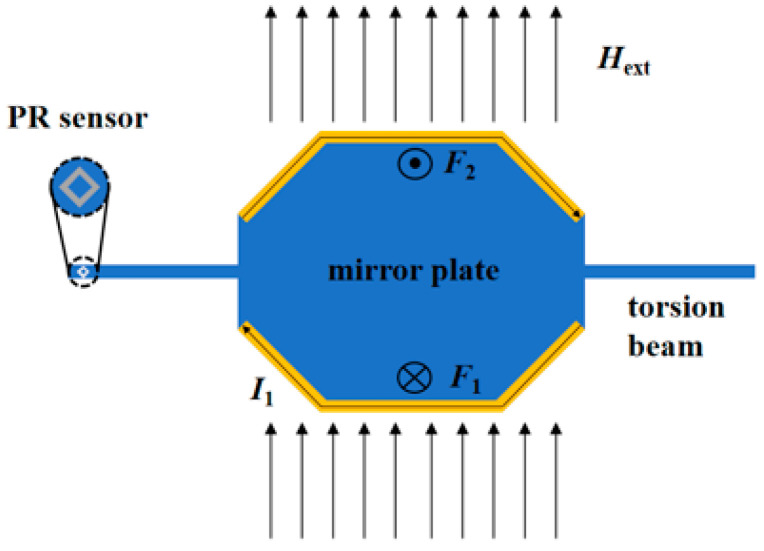
The electromagnetic micro mirror driven by a sinusoidal voltage excitation in the static magnetic field.

**Figure 3:**
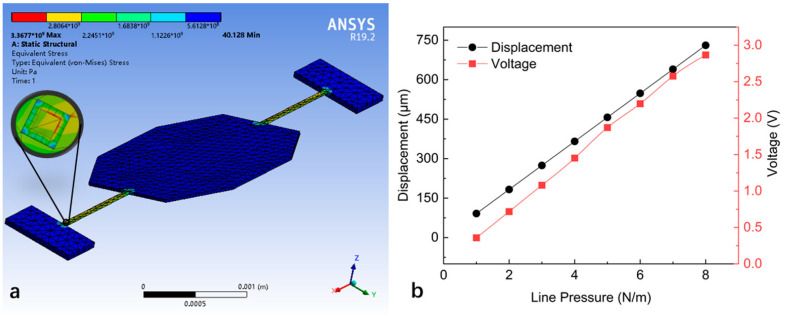
The finite element simulation of the electromagnetic micro mirror with an integrated PR sensor in ANSYS: (**a**) the model of the electromagnetic micro mirror with an integrated PR sensor; (**b**) the torsion displacement and voltage output simulated in the mechanical–piezoresistive coupling field analysis.

Meanwhile, the proposed MEMS micro mirror consists of two torsion beams, a mirror plate, and an integrated piezoresistive sensor (PR sensor), as shown in Figure 2. The designed micro mirror is supported by straight beams. When the current signal is driven to the metal coils surrounding the mirror plate, a Lorentz force is generated by the interaction of external magnetic field and the current. The micro mirror is described as a damped spring-mass system, and the dynamic equation for the linear torsion micro mirror is

(1) $$I_{m}\ddot{\theta }+c\dot{\theta }+k\theta =T_{or}(t),$$

where $I_{m}$ is the moment of the micro mirror plate’s inertia, c is the damping factor, k is the torsional beam coefficient, θ is the torsion angle, and $T_{or}$ is the torque. The Lorentz force is used as the driving force, so the torque is expressed as

(2) $$T_{or}\left(t\right)=BI\left(t\right)Lr,$$

where B is the external magnitude of the magnetic field component perpendicular to the mirror, I is the current, L is the effective length of the coil, and r is the length of the force arm. According to Equations (1) and (2), the transfer function of the micro mirror obtained by the Laplace transform is

(3) $$F\left(s\right)=\frac{1/I_{m}}{s^{2}+2\xi \omega s+\omega^{2}},$$

where s is the complex frequency, ω is the angular frequency of the micro mirror, and ξ is the damping ratio of the micro mirror. The PR sensor was fabricated on the beam of the micro mirror via P-type doping. The Wheatstone bridge is used in the PR sensor because it is most sensitive to small fractional impedance changes in one of its arms. The electrical signal was generated by the PR sensor with the change in the torsion angle of the micro mirror. Figure 3 shows the model of a micro mirror with a torsional mode of 4000 Hz and the performance of the PR sensor under external loads established by ANSYS Workbench. The long axis radius, short axis radius, and thickness of micro mirror are 1039 μm, 800 μm, and 30 μm, respectively. The length and width of the torsion beam are 750 μm and 31 μm, respectively. The output voltage of the PR sensor is linear, with displacement as a linear gain of approximately 3 V/rad.

In 1954, Charles [24] first discovered the piezoresistive effect of semiconductors such as silicon and germanium. The difference in resistance is linearly related to the applied strain, and the rate can be expressed as

(4) $$\frac{∆R}{R}=G_{1}\varepsilon ,$$

where $G_{1}$ is the strain coefficient of piezoresistance, and ε is the strain.

**Figure 4:**
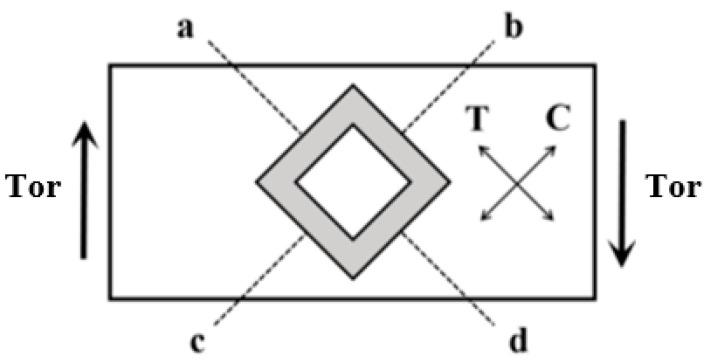
Schematic diagram of the structure of PR sensor.

The proposed PR sensor was a silicon PR sensor with a Wheatstone bridge structure, as shown in Figure 4. The PR sensor was integrated on a square torsion beam structure. When the micro mirror flipped, the torque caused the torsion of the fixed beam, resulting in tangential tensile stress T and compressive stress C on the surface of the beam. One pair of bridges was subjected to compressive stress, resulting in a decrease in resistance, while the resistance of the other pair of bridges increased due to tensile stress. The relative change in the resistance value of the bridge changed the partial voltage of the resistance. The potential difference can be described as

(5) $$V_{out}=V_{s}G_{1}\varepsilon ,$$

where $V_{out}$ is the potential difference, and $V_{s}$ is the driving voltage of the Wheatstone bridge. The output of the PR sensor has a linear relationship with mechanical force, making it sensitive to torsional motion.

**Figure 5:**
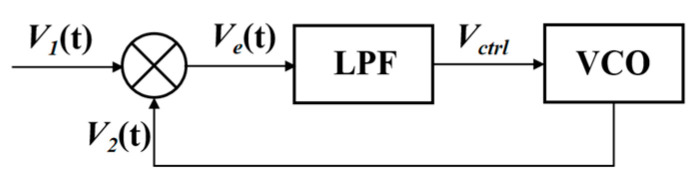
The simple scheme of the PLL applicated in the driving circuit.

Figure 5 shows the scheme of the PLL circuit, which consists of PD, LPF, and VCO. An equivalent mathematical model was established for the driving circuit based on a PLL circuit. An exclusive XOR network is used as the PD to compare the frequency of the feedback signal *V*_1_(t) with the frequency of the VCO signal *V*_2_(t) and generate an error voltage *V*_e_(t). *V*_e_(t) is filtered by the three-order LPF and applied to the control input of the VCO. The VCO with an initial frequency of 3500 Hz drives the micro mirror. The transfer function of the third-order LPF is described as

(6) $$F_{LPF}\left(s\right)=\frac{1}{\left(sR_{1}C_{1}+1\right)\left(sR_{2}C_{2}+1\right)\left(sR_{3}C_{3}+1\right)},$$

where $R_{1}$, $R_{2}$, and $R_{3}$ are the resistances in LPF, and $C_{1}$, $C_{2}$, and $C_{3}$ are the capacitances in LPF. The frequency of VCO can be described as

(7) $$f_{vco}=f_{0}+K_{vco}V_{ctrl},$$

where $f_{vco}$ is the output frequency of the VCO, $f_{0}$ is the initial frequency of the VCO, $K_{vco}$ is the frequency gain of the VCO, and $V_{ctrl}$ is the control voltage generated by the PD and LPF. In the driving circuit, the connection between the micro mirror device and the PLL circuit was realized by the waveform conversion circuits.

## 3. Simulation and Experiment

**Figure 6:**
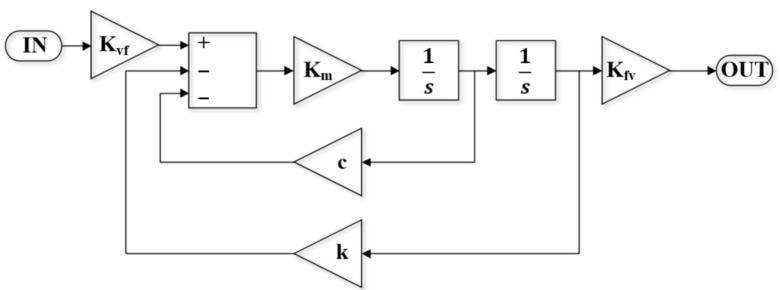
The single-degree-of-freedom dynamic model of micro mirror with PR sensor established in MATLAB Simulink.

As shown in Figure 6, the model of the micro mirror with an integrated PR sensor was established in MATLAB Simulink according to Equation (1), where K*_vf_* is the transfer function from driving voltage to torque, K*_fv_* is the transfer function of the PR sensor, K_m_ is the reciprocal of the moment of inertia *I*_m_, c is the damping factor of the micro mirror, and k is the torsional beam coefficient. The input is an excitation signal from VCO, while the output is to transmit torque to the PR sensor. We set the damping of the micro mirror to 0.001 and 1.25, respectively, to simulate the damping in vacuum and air. K*_vf_* = 1.3 × 10^−8^ N·m/V describes the equivalent torque coefficient of a micro mirror with a coil resistance of 50 Ω under a 100 mT magnetic field, and the PR sensor transfer function which converts strain into electrical signal is 3.

**Figure 7:**
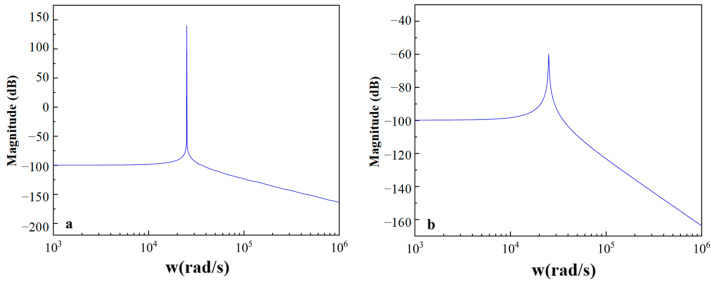
The bode plots of the micro mirrors established in MATLAB Simulink (**a**) the magnitude gains of the vacuum micro mirror; (**b**) the magnitude gains of the micro mirror in the air.

The micro mirror has a natural frequency of 4000 Hz. The micro mirror in the air has a finite Q, and the vacuum micro mirror has an infinite Q. The frequency response analysis of the micro mirror was performed, and the bode plots are depicted in Figure 7. As shown in Figure 7a,b, the micro mirror in vacuum has a higher frequency response peak and narrower bandwidth with fewer damping effects. It can be seen that micro mirrors with higher quality factors can achieve higher resonance gain to achieve a larger range of torsion angles.

**Figure 8:**
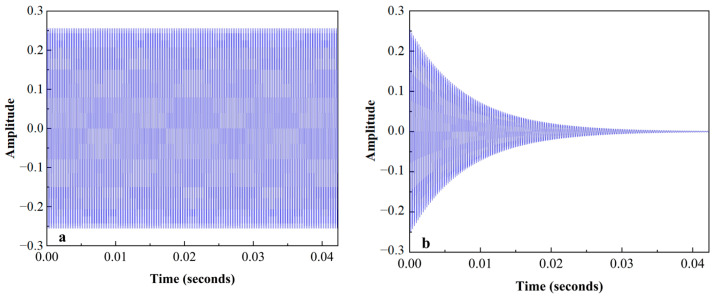
The impulse response of the micro mirrors: (**a**) the impulse response of the model with infinite Q-factor; (**b**) the impulse response of the model with a Q-factor of 100.

**Figure 9:**
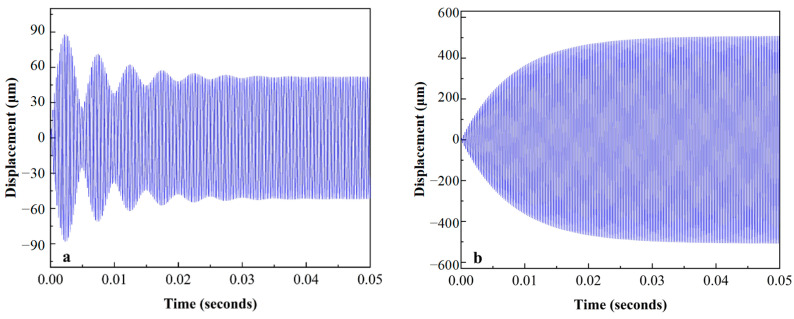
The analysis of the forced vibration: (**a**) the displacement for the micro mirror excited by a sinusoidal voltage of 3800 Hz; (**b**) the displacement for the micro mirror excited by a sinusoidal voltage of 4000 Hz.

The impulse response of the micro mirror was simulated by the model linearization. As shown in Figure 8, the model exhibited different movements under different conditions after the action of the same pulse. The micro mirror with infinite Q-factor oscillated continuously at the natural frequency, but the one with finite Q-factor gradually stopped oscillating within 0.0423 s due to damping. Therefore, external force compensation was needed for a continuous oscillation to drive the micro mirror. The Lorentz force, which was excited by a sinusoidal voltage of 3800 Hz with an amplitude of 1.5 V and a magnetic field of 100 mT, was applied to the micro mirror model, and the vibration with a maximum displacement of 52.09 μm stabilizes at about 0.043 s, as depicted in Figure 9a. However, the maximum displacement of the model was up to 488.4 μm when excited by a sinusoidal voltage of 4000 Hz with the same amplitude in Figure 9b. The maximum displacement is the torsional displacement at the edges of the micro mirror, which is linearly related to the torsion angle θ. The relationship can be expressed as follows

(8) l=r∗θ,

where r is the short axis radius.

When the excitation was applied with the natural frequency of the micro mirror, the device resonated, and the gain reached the maximum. However, if the natural frequency of the micro mirror is unknown, there is a certain error between the initial input frequency and the natural frequency. In addition, during the operation of the micro mirror, the reduction of twisting displacement was unavoidable due to the shift of frequency. A driving circuit was designed to keep the micro mirror resonant.

**Figure 10:**
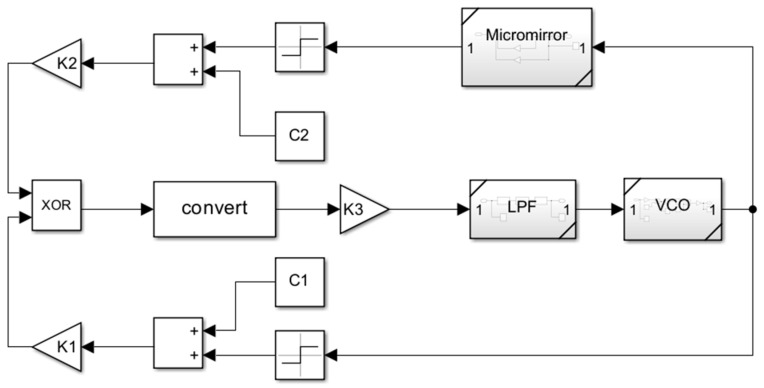
The behavior model of the micro mirror driving system established in MATLAB Simulink.

**Figure 11:**
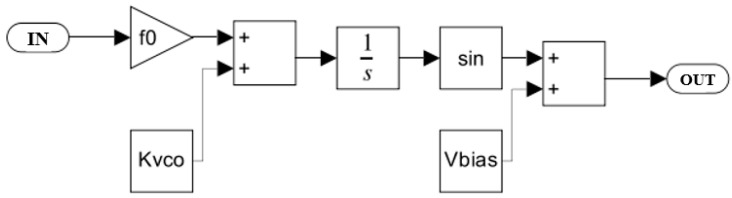
Voltage-controlled oscillator model established in MATLAB Simulink.

**Figure 12:**
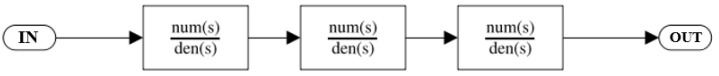
Low pass filter model established in MATLAB Simulink.

The behavior-level model of the micro mirror system was established in MATLAB Simulink, as shown in Figure 10. XOR networks are used as PDs to compare the output of VCO with the feedback of micro mirror plates. The subsystems’ performance of the micro mirror, LPF, and VCO were established, respectively. The block diagram of VCO and LPF are shown in Figure 11 and Figure 12, as described in Equations (5) and (6). LPF is a third-order LPF that filters the error signal and transmits it to VCO. The ideal VCO output is a sine wave, and its output frequency is completely linearized with the input voltage. The part from XOR to VCO implements the function of phase-locked loop. When VCO excites the micro mirror at the initial frequency, the signal frequency fed back by the PR sensor of the micro mirror will shift towards the resonant frequency. The difference between feedback frequency and excitation frequency will be captured by a phase-locked loop and tracked until it reaches the resonant frequency point and stabilizes, achieving resonant driving.

**Figure 13:**
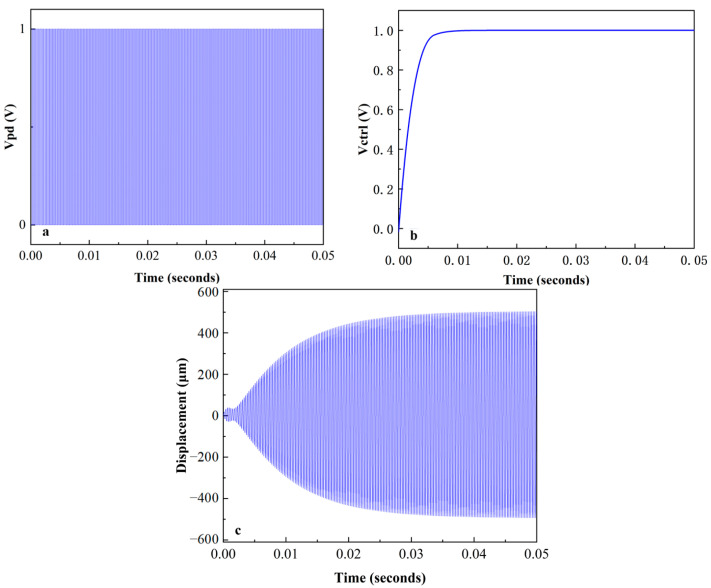
The result plots in MATLAB Simulink: (**a**) the square wave generated by the PD; (**b**) the control voltage filtered by LPF for the VCO; (**c**) the twisting displacement of the micro mirror.

The micro mirror with a Q-factor of 100 achieved self-oscillation under this system with maximum gain. The VCO began to oscillate at an initial frequency of 3500 Hz, and there was a phase difference between the micro mirror motion signal and the driving signal. As shown in Figure 13a, the difference was amplified by PD, and a square wave with a constant frequency was generated. DC voltage was generated by a third-order LPF to regulate the output frequency of VCO. The magnitude is related to the phase difference, as shown in Figure 13b, and $V_{ctrl}$ almost stabilized at 1 V after about 5 ms. The micro mirror maintained a frequency of 4000 Hz and continued to oscillate, as depicted in Figure 13c, after about 0.043 s. The PLL circuit realized the frequency tracking of the micro mirror, and the gain was similar to that of the open-loop drive simulation before. Self-oscillation was realized in the simulation of the micro mirror system, so it was possible to use an analog PLL circuit to implement a stable driving of micro mirrors.

**Figure 14:**
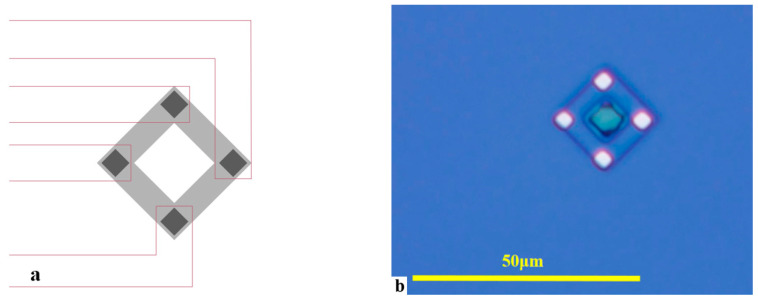
(**a**) Schematic diagram of PR sensor; (**b**) lithographic PR sensor under metallographic microscope (50×).

The PR sensor plays a feedback role in this system. As shown in Figure 14a, a silicon PR sensor with a Wheatstone bridge structure was designed, integrated at the end of the micro mirror torsion beam to measure the torsion angle of the micro mirror. A P-type Wheatstone bridge structure PR sensor with longitudinal resistance along the <110> crystal direction was fabricated on an N-type silicon wafer with a <100> crystal plane, and the length direction of the torsion beam of the micro mirror was along the <100> crystal direction. Among them, each piezo resistor forms an angle of 45° or 135° between the longitudinal direction and the long axis of the torsion beam. We designed a single bridge with a length and width of 15 μm and 3 μm, respectively. Electrodes at the four corners of the square structure were led out. Figure 14b is a graphic of the PR sensor section under a metallographic microscope.

**Figure 15:**
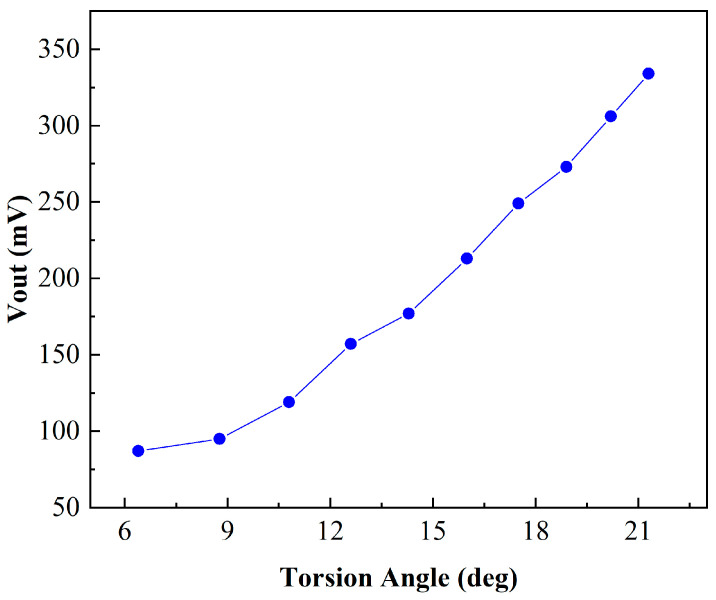
Measured PR sensor output voltage.

The working characteristic tests of the PR sensor took place, wherein an external force was applied to twist the torsion beam of the integrated PR sensor, with a 5 V power supply voltage applying to both ends of the piezoresistive bridge. The voltage amplitude of the integrated PR sensor is shown in Figure 15. It indicates that the output voltage increases linearly with the torsion angle of the micro mirror approximately. The maximum rate of the output Vpp reached 24.45 mV/deg. Since PR sensors only contain piezoresistive units without capacitors or inductors, the phase difference caused is very small. Thus, the torsion angle detection of the micro mirror is realized through the PR sensor.

**Figure 16:**
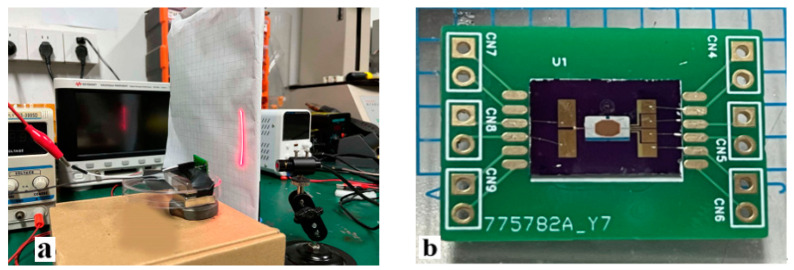
Experiment: (**a**) micro mirror testing system; (**b**) micro mirror PCB.

Finally, the effectiveness of the driving circuit was verified through measurement. Figure 16 shows the testing system and the micro mirror. The bias magnetic field was provided by the bottom permanent magnet, generating a Lorentz force to drive the micro mirror. The red laser was conducted on the mirror surface, and the reflected light spot shows the deflection of the micro mirror. The PR sensor is integrated at the end of the micro mirror torsion beam, collecting information on the flipping state of the micro mirror and providing feedback to the driving circuit.

**Figure 17:**
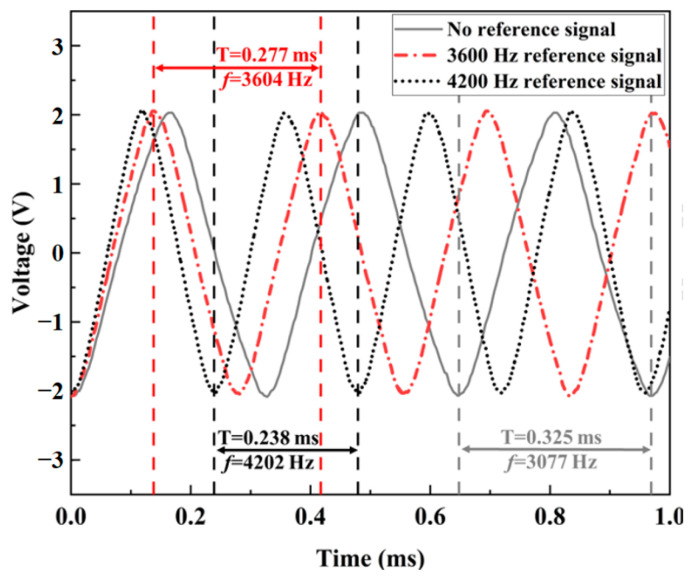
The plot of output voltage of the driving circuit.

The frequency tracking of the micro mirror system is demonstrated in Figure 17. The frequency of the sinusoidal output voltage varies with the frequency of the reference signal. When there is no reference signal, the output signal is the same as the input and is a 3077 Hz sine wave, as illustrated in Figure 17. Otherwise, the frequency of the output voltage after stabilization follows the reference signal, from 3077 Hz to 3604 Hz or 4202 Hz, with a reference frequency of 3600 Hz and 4200 Hz, respectively. There was a slight deviation between the frequency of the output signal and the reference signal, approximately no more than 10 Hz. This verifies the function of the circuit tracking frequency in this system.

**Figure 18:**
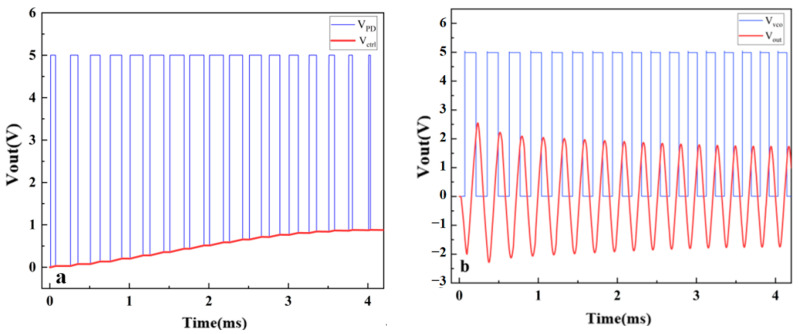
(**a**) The process of gradually changing the PD output and $V_{ctrl}$; (**b**) the process of gradually changing the output signal and excitation signal.

Figure 18a shows the changes in PD output and $V_{ctrl}$. The PD adjusts the pulse width based on the phase difference of the input signals, corresponding to the $V_{ctrl}$ of different amplitudes. Within 1–4 ms, the VCO frequency gradually increased, reducing the phase and frequency difference with the input signal. After about 4 ms, the pulse width stabilized and $V_{ctrl}$ almost remained at 0.968 V. The results are basically consistent with the simulation results in Figure 13. Figure 18b shows the process of gradually changing the output voltage from 3077 Hz to 4202 Hz. The blue line represents the output of the VCO, while the red line represents the output of the VCO after passing through a waveform converter, which is the driving signal of the micro mirror. The VCO frequency gradually increased within 1–4 ms, reducing the frequency and phase difference with the input signal. The output signal was basically stable after 4 ms, and the amplitude remained at 1.7 V. In addition, after testing, the self-excited oscillation system had a delay of approximately 14.3 ms after frequency locking stability. It is proven that the presented model coincides with the frequency tracking of PLL circuit, which confirms the validity of the proposed driving circuit. It reduces the complexity of the system and the occupied chip area and confirms the possibility of achieving low-power and lightweight design.

## 4. Conclusions

This work proposes a driving system for an electromagnetic micro mirror. The self-oscillation mode of the system was demonstrated via system-level modeling, which provides a basis for the research of high-performance electromagnetic micro mirror chips in LiDAR. An integrated PR sensor was used to achieve torsion angle detection, and it has good sensitivity and linearity, with a maximum rate of 24.45 mV/deg. The PR sensor is integrated at the end of the micro mirror torsion beam, without external connections as in [20], greatly saving space and overall complexity. The PLL circuit implemented continuous resonance in the driving and frequency tracking of the system. The micro mirror system solved the problem of instability of an open-loop drive with a compact circuit. Meanwhile, the complexity and occupied chip area of the system were reduced compared to ADC or FPGA. This possibility was confirmed to realize low-power consumption and lightweight designs. Solid-state LiDAR system resources were saved, while high-speed and high-precision scanning was realized due to the replacement of the mechanical scanning structure with micro mirrors. These advantages contribute to the research and development of LiDAR in application scenarios such as environmental detection and unmanned driving. Due to self-oscillation, the system achieved a stable scanning of 4000 Hz and ±37.6° in simulation, greatly improving the flipping angle and scanning frequency compared to [13,14]. Frequency tracking was achieved with an error of within 10 Hz in the experiment to ensure micro mirror resonance. This provides a basis for further research into high-performance LiDAR micro mirror chips. The effectiveness of the micro mirror system was verified in this paper.

## Data Availability

The original contributions presented in the study are included in the article, further inquiries can be directed to the corresponding author.
